# Removal of Organics with Ion-Exchange Resins (IEX) from Reverse Osmosis Concentrate

**DOI:** 10.3390/membranes13020136

**Published:** 2023-01-20

**Authors:** Sukanyah Devaisy, Jaya Kandasamy, Rupak Aryal, Md Abu Hasan Johir, Harsha Ratnaweera, Saravanamuthu Vigneswaran

**Affiliations:** 1 Faculty of Engineering, University of Technology Sydney (UTS), Broadway, NSW 2007, Australia; 2 Department of Bio-Science, Faculty of Applied Science, University of Vavuniya, Vavuniya 43000, Sri Lanka; 3 Faculty of Sciences and Technology (RealTek), Norwegian University of Life Sciences, NO-1432 Ås, Norway

**Keywords:** reverse osmosis concentrate, dissolved organics, micro-pollutants, adsorption, ion-exchange resin, submerged membrane system

## Abstract

Reverse osmosis concentrate (ROC) produced as the by-product of the reverse osmosis process consists of a high load of organics (macro and micro) that potentially cause eco-toxicological effects in the environment. Previous studies focused on the removal of such compounds using oxidation, adsorption, and membrane-based treatments. However, these methods were not always efficient and formed toxic by-products. The impact of ion-exchange resin (IEX) (Purolite^®^A502PS) was studied in a micro-filtration–IEX hybrid system to remove organics from ROC for varying doses of Purolite^®^ A502PS (5–20 g/L) at a flux of 36 L/m^2^h. The purolite particles in the membrane reactor reduced membrane fouling, evidenced by the reduction of transmembrane pressure (TMP), by pre-adsorbing the organics, and by mechanically scouring the membrane. The dissolved organic carbon was reduced by 45–60%, out of which 48–81% of the hydrophilics were removed followed by the hydrophobics and low molecular weight compounds (LMWs). This was based on fluorescence excitation-emission matrix and liquid chromatography-organic carbon detection. Negatively charged and hydrophobic organic compounds were preferentially removed by resin. Long-term experiments with different daily replacements of resin are suggested to minimize the resin requirements and energy consumption.

## 1. Introduction

Reverse osmosis (RO) is an emerging water treatment technology that satisfies the growing freshwater demand for the global population. RO plants operate with water recoveries of between 35 and 85% and consequently generate huge volumes of reverse osmosis concentrate (ROC) as a by-product [1]. ROC contains all the rejected compounds such as salts, dissolved organics, and pharmaceuticals and personal care products (PPCPs) [2], and the discharge of these into inland/marine water bodies will likely cause adverse eco-toxicological effects and threaten aquatic ecosystem [1]. 

Detailed investigation on organic fraction reveals that ROC contains 83–90% hydrophilics which comprises mostly humic substances (72–76%) [3]. In recent years, fluorescence spectroscopy has been suggested as a reliable optical technique for monitoring organic matter in water and wastewater [4]. Fluorescence measurements, called an excitation emission matrix (EEM), are rapid and highly sensitive, need no reagents, and require minimum sample pretreatment processes [5]. The EEM can provide a range of chemicals such as humic-like, fulvic-like, biopolymers, aromatic proteins, etc., present in dissolved organic matter in water and/or wastewater. Fluorescence excitation-emission matrix (FEEM) analysis results reported in a previous paper [6] confirmed that ROC contains more hydrophilic products such as humic acid and fulvic acid. The above-mentioned hydrophilic substances were found to adversely impact the environment by forming disinfection by-products [7], stimulating the growth of dinoflagellate, which is an algal bloom in the coastal environment [8], influencing the transport and the redox state of metal ions [9], and mobilizing pesticides and other contaminants in the environment, thereby leading to greater persistence of contaminants in the environment [9]. Therefore, it is vital to explore a suitable treatment system for ROC for the effective abatement of organics by targeting the preferential removal of hydrophilic substances. 

Since the organics in the ROC are not so biodegradable (BOD/COD ratio is 0.24 and below 0.30) [6], there is a need to explore physicochemical treatment techniques to remove them. There are several advanced treatment technologies that have been investigated before such as oxidation, coagulation, carbon adsorption, ozonation, ion exchange resin, etc. [3,6,10,11]. The coagulation process removed hydrophobics (49%) more than transphilics (27%) and hydrophilics (12%), whilst combined coagulation–UV/H_2_O_2_ oxidation removed these at 87%, 70%, and 39%, respectively [12]. The combined ozonation and biological activated carbon process removed biopolymers (11%), building blocks (35%), and LMW neutrals. GAC was found to be more selective towards hydrophobic organic substances [13]. 

Jamil et al. [3,14] reported that the removal of DOC with GAC adsorption was larger compared to strong base anion exchange resins (Purolite^®^ A502PS, Purolite^®^ A860S). However, the anion exchange resins (AER) were found to remove more hydrophilic fractions selectively from DOC [15]. A study by Fan et al. [16] showed that a novel magnetic anion exchange resin (NDMP) removed more hydrophilics and low molecular weight fractions (<3 kDa) from dye bio-treatment effluents, whilst particle active carbon achieved lower DOC removals (15%). A fluidized bed packed with an anion exchange resin (Purolite^®^ A500P) was found to remove 76.4% of hydrophilics and 55% of hydrophobics from synthetic wastewater [17]. 

Ion exchange is a reversible process which exchanges ions between solid and liquid phases. The solid phase is the ion exchanger resin which carries exchangeable ions, and the liquid phase contains the electrolytes. The ion-exchange resin does not undergo structural changes during the reaction [18]. The cation and anion exchange reactions generally occur as shown below (Equations (1) and (2)):

(1) $$2\;\mathrm{Na}-\mathrm{Resin}+{\mathrm{CaCl}}_{2\;\left(\mathrm{aq}\right)}↔\;\mathrm{Ca}-{\mathrm{Resin}}_{2}+2{\mathrm{NaCl}}_{\left(\mathrm{aq}\right)}$$

(2) $$2\;\mathrm{Resin}-\mathrm{Cl}+{\;\mathrm{Na}}_{2}{\mathrm{SO}}_{4\;\left(\mathrm{aq}\right)}↔{\;\mathrm{Resin}}_{2}-{\mathrm{SO}}_{4}+2{\;\mathrm{NaCl}}_{\left(\mathrm{aq}\right)}$$

The reaction shown in Equation (1) is attributed to the softening of water. When hard water (water with Ca ions) is pumped through a column packed with anion-exchange resin (Na-Resin), the calcium ions are removed from the water and replaced by an equivalent amount of sodium. Once all the sodium ions in the resin are replaced, the resin will become ‘exhausted’, which may be regenerated with NaCl solution [18]. 

As per previous studies, strong basic anion-exchange resins were found to be better at removing hydrophilic organics (especially humic substances and fulvic acids) from DOC and inorganic anions from feed water [7,19]. This could reduce membrane scaling in the subsequent steps of an experiment [20]. Strong basic anion-exchange resin, which includes quaternary ammonium resins in chlorine form, was used in organics removal [7] which can be represented by the following reaction, where the charged DOC is represented by $R^{-}$:

(3) $$\mathrm{Resin}-{\mathrm{NMe}}_{3}^{+}{\mathrm{Cl}}^{-}+R^{-}↔\mathrm{Resin}-{\mathrm{NMe}}_{3}^{+}R^{-}+{\mathrm{Cl}}^{-}$$

In addition to the removal of hydrophilic substances, the strong basic anion-exchange resins were found to remove acidic and negatively charged synthetic organic micro-pollutants through electrostatic interactions [21,22] between the carboxylic acid group and strong-base functional groups in conjunction with the non-electrostatic interaction between the benzene rings of some pharmaceuticals and polystyrene polymer matrix [22]. 

Previous researchers have used several techniques to remove DOC from the ROC; however, few address the removal mechanism of hydrophilic organics from DOC in ROC using ion-exchange resins. This preliminary study was conducted to assess the feasibility of using a strong basic anion-exchange resin with an MF membrane to remove hydrophilic substances in an MF–IEX hybrid system. Here, the MF was used to retain the fine resin particles entering the treated water. A detailed characterization of dissolved organic matter and organic micro-pollutants in the ROC and treated ROC was made to study the removal mechanism of organics using ion-exchange resins.

## 2. Materials and Methods

### 2.1. Materials

#### 2.1.1. Wastewater

ROC water samples collected from a wastewater treatment plant (WTP) in Sydney, Australia were used as the feed water for the experiment. In the WTP, biologically treated sewage effluent was further treated to form recycled water using a dual membrane process employing micro-filtration (0.2 µm) and RO. The recycled water is supplied back to the consumers living in the surrounding suburbs for non-potable purposes. By doing so, more than 800 million litres of potable water annually was saved by not using it for non-drinking purposes [23]. ROC is the only waste generated from the RO filters at the WTP where approximately 300 kL of ROC is produced per day [24]. Currently, the WTP does not treat the ROC, and it is discharged to the sewer [23].

#### 2.1.2. Ion Exchange Resin (IEX)

Purolite^®^ A502PS, a commercially available anion-exchange resin (IEX), was used in combination with an MF membrane. The IEX is a polystyrenic Macroporous, Type I Strong Base Anion Resin which is a Type 1, quaternary/ammonium with chloride ion. Its total exchange capacity (min) is 0.85 eq/L (18.6 kg/ft^3^), and its moisture retention is 66–72%. Particle sizes ranging between 425 and 600 µm were selected for this experiment. The authors found in their previous study that this range of particle sizes was effective in reducing the transmembrane pressure (TMP) of the submerged membrane hybrid system [19]. 

#### 2.1.3. MF Membrane 

The characteristics of the hollow fibre membranes (MANN+HUMMEL ULTRA-FLO PTE LTD, Singapore) used in the MF–IEX hybrid system are given in Table 1. Purolite^®^ A502PS was used in combination with an MF membrane. 

### 2.2. Methods 

#### 2.2.1. Membrane–IEX Hybrid System 

**Figure 1:**
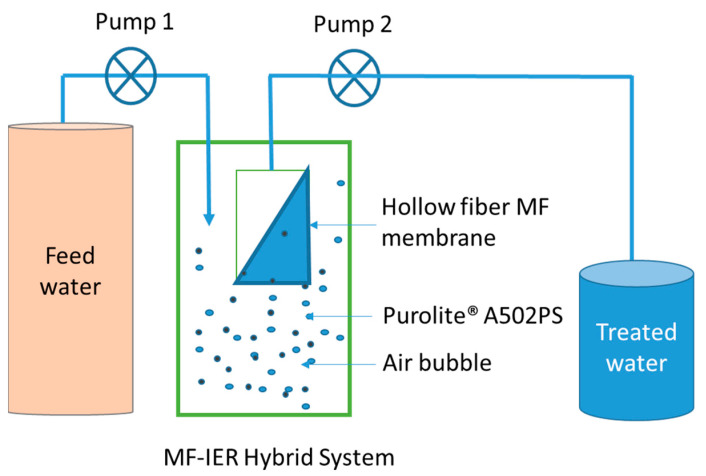
Membrane–IEX Hybrid System (Dose of Purolite^®^ A502PS is 5 g/L, 10 g/L, and 20 g/L; flow rate is 36 L/m^2^·h).

The effect of Purolite^®^ A502PS IEX on the removal of dissolved organics (DOC) and the organic micro-pollutants was studied using an MF–IEX hybrid system (Figure 1). A hollow fibre MF membrane was submerged in the reactor tank containing 3 L of ROC. The flow of influent (ROC) and effluent (treated water) was controlled using two master flux peristaltic pumps. The TMP of the membrane filtration was measured using a pressure gauge. Different doses of Purolite^®^ A502PS (5 g/L, 10 g/L; 20 g/L) were added to the reactor tank. The flux of influent and effluent was 36 L/m^2^·h which maintained a constant water level in the reactor. The reactor tank was fed with continuous air flow at 1.5 m^3^/m^2^ membrane area h (pre-determined) to keep the Purolite^®^ A502PS particles in suspension to enhance the removal of contaminants. The hybrid system was found to be effective for two reasons. First, because the prior removal of organics/other charged compounds before they reach the membrane surface reduced fouling/scaling effects on the membrane surface, and second, because the airflow produces shear stress across the membrane surface, and its scouring effect further reduces the deposition of organics and reduces fouling [25,26]. 

The loss of volatile organic compounds (VOCs) due to aeration was neglected as the wastewater used in this study was previously biologically treated and stabilized. The primary purpose of hollow fibre MF was to remove tiny purolite particles, if any, from treated water. The TMP of the MF–IEX hybrid system was measured using a pressure gauge (Novus log box). 

The MF membrane alone can remove less than 10% of the DOC from the wastewater due to the larger pore size, which is not small enough to retain organic molecules [26]. Purolite^®^A502PS was added to enhance the removal of organics from the ROC in the MF–IEX hybrid system. The authors’ previous study [19], reported that 1 g/L of Purolite^®^ A502PS was optimum in removing organics from RO feed using pre-adsorption of organics. Since ROC is ~5 times more concentrated than RO feed, a 5-fold increase in Purolite^®^ A502PS dosage (5 g/L) was used in MF–IEX hybrid system to achieve optimum organic removal. Further, higher doses (10–20 g/L), were also trialled to enhance the removal of micro-pollutants so as to overcome the competitive effect of the organics for Purolite^®^ A502PS exchange sites.

In addition, the authors have performed a similar short-term experiment with GAC at varying dosages of 5 g/L, 10 g/L, and 20 g/L using the ROC as the feed for the same experimental conditions. The respective DOC removals were observed to be 20–50%, 60–80%, and 70–90% over 4 h of operation [26]. Though GAC was found to reduce the organic load in several studies, it also reduced the sites available for sorption and removal of other micro- or priority organic pollutants [27]. Further, the removal of humics with ion-exchange resin (Purolite) is excellent compared to GAC [3]. In this context, the performance of Purolite^®^A502PS in the MF–IEX hybrid system was studied for the removal of organic fractions and organic micro-pollutants at the dosage of 5 g/L, 10 g/L, and 20 g/L. The ion-exchange resin was added only at the start of the experiment and no further additions were made during the experimental run.

#### 2.2.2. Dissolved Organic Carbon (DOC) 

The water samples collected from the membrane–IEX hybrid were filtered with 0.45 µm filter paper and analysed for DOC using a Multi N/C 2000 analyser (Analytik Jena AG, Jena, Germany). The samples were injected into the system using an autosampler, and the samples were automatically analysed for total carbon (TC) and inorganic carbon (IC). The DOC was determined using the difference between the TC and IC. The calibration was regularly done using standard glucose solutions. 

#### 2.2.3. Liquid Chromatography-Organic Carbon Detection (LC-OCD) 

LC-OCD is a method that separates the pool of natural organic matter into major fractions based on the size of molecules and then quantifies these compounds on the basis of organic compounds [28]. The LC-OCD system consists of a size exclusion chromatography column, which separates hydrophilic organic molecules according to their molecular weight size. The separated organic compounds were then detected using two different detectors: a UV detector (absorption at 254 nm) and a DOC detector (after inorganic carbon purging). The different classes of organic matter can be identified quantitatively and qualitatively depending on the size of the molecules. The column has a separation range of 0.1–10 kDa [28]. In this regard, Model 8 developed by DOC Labor, Dr Huber (Germany) served to characterise the organic compounds in detail. A Toyopearl TSK HW50S column (TOSOH Bioscience GmbH, Stuttgart, Germany) was used with a phosphate buffer mobile phase of pH 6.4 (2.6 g/L KH_2_PO_4_ and 1.5 mol/L Na_2_HPO_4_) at a flow rate of 1.1 mL/min. Injection volumes and retention time were set at 1000 μL and 120 min, respectively. The chromatographic column was a weak cation-exchange column based on polymethacrylate.

Natural organic matter can be categorised into two major types, i.e., hydrophobics and hydrophilics. The hydrophilics can be further sub-divided into biopolymers, humics, building blocks (weathering products of humic substances), low molecular weight (LMW) neutrals, and LMW acids. The wastewater filtered with MF consists mainly of humics (~50%). The derivatives of humics are called building blocks which were also found significantly in large amounts. As the wastewater was collected just after MF, the biopolymers were low as they had been captured with the MF process. The LMWs as well were quite low as they had already been consumed by bacteria at the biological treatment stage. 

#### 2.2.4. Fluorescence Excitation-Emission Matrix (FEEM) 

FEEMs were measured using a Varian Eclipse fluorescence spectrophotometer (Varian Cary Eclipse Fluorescence Spectrophotometer, Waltham, MA, USA). The 3D-EEM technique is a rapid, selective, sensitive, and informative way to characterize a number of groups of chemicals of interest. The EEM generates information regarding the fluorescence characteristics of organic compounds by simultaneously changing the excitation and emission wavelengths. The fluorescence in different spectral regions is associated with various types of functional groups. The fluorescence signals of DOM in water and wastewater are basically attributed to protein-like fluorophores, fulvic-like fluorophores, and humic-like fluorophores, and they characterize dissolved organic matter in water using fluorescence spectroscopy as shown in Figure S1 and Table S1 [29]. EEMs were recorded using scanning emission wavelengths from 250 to 500 nm repeatedly at excitation wavelengths scanned from 220 to 400 nm in 5 nm increments. The excitation and emission bandwidths were both set at 5 nm. The fluorometer was set at a speed of 3000 nm/min, a PMT voltage of 700 V, and a response time of 2 s. The blank solution (ultrapure water) was scanned and deducted from the original sample for blank correction.

#### 2.2.5. Liquid Chromatography-Mass Spectrometry (LC-MS) 

Micro-pollutants were extracted using solid phase extraction (SPE) and examined employing liquid chromatography with tandem mass spectroscopy. A total of 5 mL analytes were extracted using 500 mg hydrophilic/lipophilic balance (HLB) cartridges (Waters, Milford, MA, USA). These analytes were separated using an Agilent (Palo Alto, CA, USA) 1200 series high performance liquid chromatography (HPLC) system equipped with a 150 × 4.6 mm, 5 µm particle size, Luna C18 (2) column (Phenomenex, Torrance, CA, USA). Mass spectrometry was conducted using an API 4000 triple quadrupole mass spectrometer (Applied Biosystems, Foster City, CA, USA) equipped with a turbo-V ion source employed in both positive and negative electro-spray modes. All calibration curves had a correlation coefficient of 0.99 or better. Details of the analysis are described elsewhere [30,31].

## 3. Results

### 3.1. Characteristics of the ROC 

The general characteristics and the inorganic cations and anions of the ROC are shown in Table 2.

The concentration of DOC was 30.8 mg/L, and its detailed fraction obtained using LC-OCD is given in Table 3. The LC-OCD separated DOC into five organic fractions i.e., biopolymers (polysaccharides, proteins, and amino sugars, MW > 20,000 gmol^–1^), humic substances (humic and fulvic acids, MW 500–1000 gmol^–1^), building blocks (hydrolysates or breakdown products of humics, MW 300–500 gmol^–1^, LMW acids (aliphatic LMW organic acids, MW < 350 gmol^–1^), and LMW neutrals (alcohols, aldehydes, ketones, sugars, and amino acids, MW < 350 gmol^–1^) [32]. A sample LC-OCD spectrum is given in Figure S2.

In the ROC, the hydrophilic fractions are comparatively higher (~89%), whilst the hydrophobics are low (~11%). Within the hydrophilic fraction, the humics content is the highest followed by LMW neutrals and building blocks. The biopolymers are negligible in quantity. Therefore, the removal of humics is essential as it comprises a major part of the organics in the ROC. 

The quantity of organic micro-pollutants in the ROC obtained using the LC-MS are presented in Table 4. The ROC sample was tested for more than 35 different types of organic micro-pollutants out of which 17 micro-pollutants, including enalapril, risperidone, linuron, atorvastatin, omeprazole, meprobamate, hydroxyzine, diazepam, diazinon, ibuprofen, sim-hydroxyacid, simvastatin, t-octylphenol, polyparaben, phenylphenol, triamterene, and atrazine, were detected below its LOQ.

Carbamazepine was detected at high concentrations (2.24 µg/L), followed by caffeine (1.41 µg/L) and trimethoprim (0.97 µg/L). The high levels of such micropollutants were found to cause adverse impacts on the aquatic ecosystems. Qiang et al. [33] reported that even an exposure to 1 μg/L carbamazepine disturbed the expression pattern of neural-related genes of zebrafish embryos and larvae. Pires et al. [34] found an increased level of lipid peroxidation (which is an indicator of oxidative stress) in two Polychaeta species after 28 days of exposure to caffeine at 0.5 μg/L. Trimethoprim is extremely persistent in the environment [35]. The rest were detected at below 0.5 µg/L; however, depending on the nature of the aquatic species, the micro-pollutants may harm their physiology. 

### 3.2. TMP Development and DOC Removal 

The TMP was developed progressively from 100 mbar to 350 mbar in the absence of Purolite^®^ A502PS over 400 min of operation. With the addition of resin, the TMP increased only to 250 mbar irrespective of the dosage. The addition of resin removed free organics from the wastewater before they could reach the membrane surface, thereby reducing membrane fouling by limiting the deposition of organics on the membrane surface [19,25]. This reduced the TMP by 100 mbar. A similar observation was reported in our previous study with the same dosages (5 g/L–20 g/L) of GAC adsorbent when used for the treatment of ROC in an MF–adsorption hybrid system [41]. This is in line with Alborzi et al. [42], who reported the lower TMP of 100 mbar led to particle accumulation on the membrane surface as the predominant fouling mechanism. 

**Figure 2:**
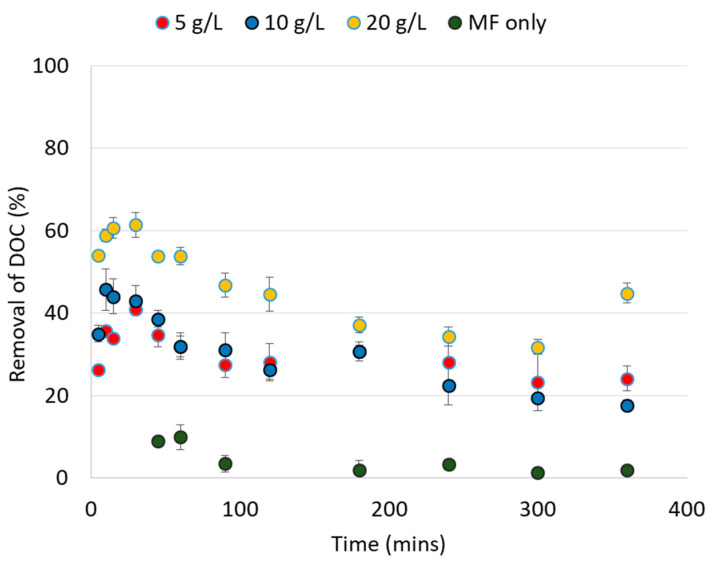
The removal of DOC with the MF–IEX hybrid system for the varying doses of Purolite^®^ A502PS (0, 5, 10, 20 g/L). Some error bars are obscured by the symbol due to its size.

This further shows the decreasing trend of DOC in the effluent, which correlates well with the reduction of TMP development (Figure 2). This may be interpreted as a reduction of membrane fouling limits the rise in TMP. However, the degree of fouling is largely dependent on the organic fractions. Lee et al. [43] reported that the high hydrophilic content of wastewater is responsible for the reduction in flux and membrane fouling in MF membranes as the macro-molecules block the pores of the MF membrane. Other previous studies reported that the membrane fouling is primarily caused by the hydrophilic content [44,45,46,47]. In this context, the measurement of organic fractions in the influent and effluent water samples are needed. 

### 3.3. FEEM Spectra 

**Figure 3:**
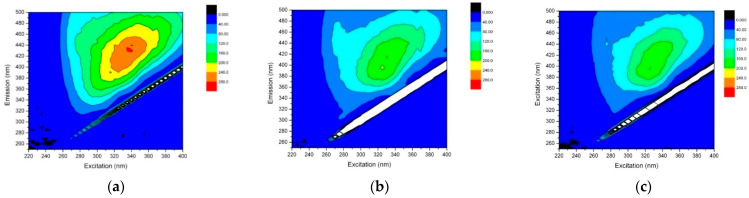
FEEM plots of organics removed with Purolite^®^ A502PS in MF–IEX hybrid system at flux 36 LMH, (**a**) ROC (**b**) Purolite dose of 10 g/L and (**c**) Purolite dose of 20 g/L.

The removal of the organic fractions was initially characterized based on its fluorogenic nature. The plots of FEEM obtained for the ROC and different doses of resin are given in Figure 3. The FEEM shows fluorescent fractions of organics which absorb light and remit light energy as fluorophores. The organics fractions are shown as a combination of peaks at the excitation wavelength (Ex) and the emission wavelength (Em). The FEEM patterns obtained are mainly due to aromatic DOC fractions in the treated water [32]. 

Figure 3a shows the FEEM of the ROC. The high intensity area of Ex/Em = 340 nm/430 nm corresponds to humic acid-like substances and fulvic acid-like organics which contain higher levels of carboxyl content [48]. Figure 3b,c corresponds to the treated water where the less intense region shows how the resin reduced the humic acid-like and fulvic acid-like compounds significantly. 

The Ex:Em appearing at 320–350:400–450 in the EEM spectra is typical for raw wastewater [49,50]. The fluoropore shows that the majority of DOC in the ROC is humic-related substances. The ROC samples were measured using UV beforehand, and absorbances were below 0.1 ABS to ensure the fluorescence testing samples would avoid the fluorescence saturation and quenching effect. The fluoropore intensity of the ROC when measured in dilution series showed linear proportional to the concentration. The Purolite^®^ A502PS at the dose of 10 g/L and 20 g /L reduced the fluorpore intensity by almost 40 and 50%, respectively, which is in-line with the LC-OCD results.

### 3.4. Determination of Organic Fractions

The different organic fraction removals with Purolite^®^ A502PS in the MF–IEX hybrid system are tabulated in Table 5. It was observed that Purolite^®^ A502PS removed hydrophilics (especially humics and building blocks) in preference to hydrophobics, and the overall removals were enhanced with the increased dosages of resin. Further, at the dosage of 10 g/L, within the hydrophilic content, the highest removal was building blocks (83.5%) followed by humics (48.2%). Building blocks are weathering products of humic substances. LMW neutrals were not significantly removed with the resin. Biopolymers were detected in the treated water at concentrations greater than in the raw ROC. This could be due to resin leaching. This is consistent with a study by Bassandeh et al. [32] where resin leaching was observed for organic fractions with MW > 20,000 gmol^−1^ which include biopolymers. This shows the degradation on the structure of resin. The increased dosage of 20 g/L also showed a similar trend of removal, though, as expected, the removal of each fraction was higher. 

The removal of organics using ion-exchange resin can be explained by two mechanisms. The first mechanism is ion exchange (electrostatic interaction) which is the release of a counter ion from the resin surface by an electrostatic interaction between functional groups of the resin and the organic matter. The ion exchange mechanism arises due to an electrostatic interaction occurring between the resin functional groups (quaternary amine groups) and the carboxylic acid moieties in the DOC. The negatively charged organic fractions such as humics and fulvic acids are predominantly removed by this mechanism [32]. The second mechanism is physical adsorption where van der Waals forces exist between the polymer backbone of the resin and the non-ionic moieties of the organic matter [51]. This may also be expressed as hydrophobic interaction. Therefore, the removal of hydrophobics fraction can be explained by physical adsorption. It was also stated by Pürschel et al. [52] that polystyrene resin provides a better organic removal and removes hydrophobic neutral fraction through π–π bonding. 

The resin dosage of 10 g/L reduced hydrophobics by 24.5%, while the increased dosage of 20 g/L improved the removal to 36.5%. The slightly enhanced removal of hydrophobic by the higher dosage of resin could be due to more available polymer backbones to adsorb organics through van der Waals forces. An experiment performed with different doses by Rahmani showed [51] that a decrease in resin dose had a lower impact on the removal of hydrophobics and a higher impact on the hydrophilic compound. The same phenomenon was also observed in this study where the increased dose of resin from 10 g/L to 20 g/L enhanced the removal of hydrophobics and hydrophilic (humics); however, the removal of the latter increased by two-fold from 48.2% to 81.2%. On the contrary, the removal of building blocks was reduced with an increased resin dosage. This may be due to some other factors influencing the removal such as solute–solvent interactions and the presence of other inorganics molecules [51]. 

In the removal of organic fractions, the MW of each fraction is another factor that could influence their removals with resins [53]. In this study, the organic fractions such as humics (48.2–82.2%) and building blocks (45.2–83.5%) were preferentially removed using Purolite^®^ A502PS compared to biopolymers (0%) and LMW neutrals (14.1–15%). This observation is in line with a study by Bazri and Mohseni [54] where lower and moderate MW organics showed higher removals compared to larger MW organics. The results obtained from this study support that the biopolymers having higher MW (>20,000 Da) were not removed with the Purolite^®^ A502PS, while the humics with moderate MW (~1000 Da) and the building blocks with small MW (300–500 Da) were preferentially removed [55]. LMW neutrals having smaller MW (>350 Da) were not significantly removed with the resin. This is consistent with the previous studies [53,56]. This could be due to the hydrophobic nature of the molecules [57] which could be interpreted as physical adsorption [32]. The MW of the organic fractions were adapted from Simon et al. [58]. 

In addition, other factors such as solvent properties, organic–solvent interaction, and the presence of other inorganic ions (nitrate and sulphate) also need to be considered to understand the removal of organics using resin [51]. The presence of inorganic ions can change the selectivity of the resin towards some specific fractions or organics. The presence of higher charged density ions (sulphate) diminishes the electrostatic interaction thereby enhancing the removal of hydrophobics with physical adsorption, while the influence of low charge density ions such as nitrate is insignificant [59]. The ROC used in this study contains a high concentration of sulphate ions (171–200 ppm) and this might have enhanced the removal of hydrophobic fractions. 

### 3.5. Organic Micro-Pollutants 

As shown in Table 6, the Purolite^®^ A502PS was found to remove organic micro-pollutants effectively from the ROC. More than 65% removal was observed with the lower dosage of 5 g/L and more than 81% removal at the 20 g/L dosage. The increase in Purolite^®^ A502PS dose enhanced the removal of micro-pollutants.

Micro-pollutants, which are usually hydrophobic and charged organic molecules having a molecular weight of less than 1000 Da, can easily be absorbed onto the highly hydrophobic and charged synthetic polymer surface [60].

Micro-pollutants such as ketoprofen, naproxen, gemfibrozil, paracetamol, sulfamethoxazole, and primidone are effectively removed by more than 77% with a dose of 5 g/L resin and more than 81% with a dose of 20 g/L. However, the removal of diclofenace with 5 g/L resin was quite low, i.e., 65%, but the removal with 20 g/L was 96%. The higher performance of such micro-pollutants could be due to a negative charge, which could have been removed through electrostatic interactions and the ion exchange mechanism [22]. Naproxen and ketoprofen showed higher removals (>90%) even at the lower dose of resin, which could be due to the higher hydrophobicity of the molecules facilitated through physical adsorption into the resin polymer [22]. Gemfibrozil and diclofenac showed relatively less removal (65–75%) at the lower dose and higher removal (>90%) with a dose of 20 g/L. This could be due to competition with other organic and inorganic ions. 

Triclocarbon, triclosan, amitriptyline, verapamil, fluoxetine, clozapine, diuron, carbamazepine, DEET, and simazine bearing either neutral or positive charge were removed by more than 71% with a dose of 5 g/L and 86% with 20 g/L. Since Purolite^®^ A502PS is an anion exchange resin, the removal of these micro-pollutants could have been achieved with physical adsorption due to their hydrophobicity (Log Kow values > 2). The increase in dose from 5 g/L to 20 g/L (four-fold) enhanced the removal only marginally. Rahmani [51] similarly found that an increase in dose had less impact on the removal of neutral/positively charged micro-pollutants. 

Atenolol, caffeine, trimethoprim, and TCEP are neutral/positively charged and less hydrophobic (Log Kow < 2). They had removals of between 66 and 93% with a dose of 5 g/L and between 82 and 98% at 20 g/L. Though they show better removals, a significant level of micro-pollutants still remained in the treated water at 114, 97, 149 and 77 ng/L, respectively, after the treatment. Solute–solvent properties, the presence of inorganic ions (nitrate and sulphates), and other factors need to be studied to address the gaps in the removal efficiencies of different types of micro-pollutants with resin. 

Although the experiments in this study were conducted for a short time to show the superiority in the removal of the humic fraction, a long-term experiment with an MF–GAC hybrid system (initial dose of 10 g/L, with 10% daily replacement) showed that it can be operated for a long period [26]. It showed good removal of organics (60–80%) and very minimum membrane fouling (minimal TMP development) which could be due to the removal of major organic foulants using the adsorbent. 

Another long-term experiment (56 days) conducted by Jeong et al. [61] showed that the addition of PAC with MBR resulted in significant fouling reduction on a membrane by adsorbing high molecular weight organics from sea water. Nguyen et al. [62] also stated that a GAC filter adsorbs humic-like, fulvic-like, and protein-like organic compounds and decreased membrane fouling potential. 

In this study, more importantly, the Purolite^®^A502PS preferentially remove hydrophilic organics, which is the major organic foulant that causes membrane fouling. Therefore, further research should focus the long-term operation of MF–IEX hybrid systems with an appropriate daily replacement of resin. 

Although an economic cost analysis has not been considered in this study, our previous study showed that Purolite^®^A502PS can be easily regenerated with 1 N NaCl solution in fluidized beds. Nur et al. [63] reported that Purolite^®^A502PS based resins (A520E) can be effectively desorbed using 1 M NaOH by maintaining the adsorption capacity at >90% of the original value. Therefore, the use of Purolite^®^A502PS in this MF–IEX hybrid system could be a cost effective subject to the appropriate regeneration

## 4. Conclusions

The MF–IEX hybrid system was effective at removing hydrophilic organics and synthetic organic micro-pollutants. Purolite^®^ A502PS significantly reduced membrane fouling evidenced through TMP development. LC-OCD showed the resin preferentially removed humic substances and building blocks efficiently using the ion exchange mechanism and the hydrophobic organics using physical sorption. The former mechanism is more efficient than the later. Resin degradation occurred, which showed a higher concentration in the effluent than the influent. The increase in dosage had less impact on the removal of hydrophobic organics. The resin was effective at removing negatively charged and high hydrophobic (LogKow > 2) synthetic micro-pollutants from the ROC. The neutral/positively charged micro-pollutants were also removed, and this could be predominantly due to physical adsorption.

## Figures and Tables

**Table 1:** Characteristics of the hollow fibre membrane.

Item	Characteristics
Material	Hydrophilic modified Polyacrylonitrile (PAN)
Nominal pore size	0.10 μm
Outer diameter	2.1 mm
Inner diameter	1.1 mm
Surface area	0.2 m^2^
Manufacturer	MANN+HUMMEL ULTRA-FLO PTE LTD, Singapore

**Table 2:** Characteristics of the ROC.

	Parameters	Unit	Level
General	Conductivity	µS	2655 ± 10
	pH		6.8 ± 0.5
	ORP	mV	41
	DOC	ppm	29 ± 3
	TDS	ppm	1950 ± 12
Inorganic anions	Fluoride	ppm	2.8 ± 0.2
	Chloride	ppm	445 ± 10
	Nitrate	ppm	38 ± 5
	Sulphate	ppm	223 ± 14
Inorganic cations	Na	ppm	450 ± 25
	K	ppm	101 ± 20
	Ca	ppm	109 ± 11
	Mg	ppm	55 ± 5

**Table 3:** Detailed fraction of organics in the ROC.

	DOC							
NOM Fraction	Total	Hydrophobic	Hydrophilic	Bio- Polymers	Humics	Building Block	LMW Neutrals	LMW Acids
Conc (mg/L)	30.8 ± 2	3.44 ± 0.9	27.34 ± 2.0	0.66 ± 0.1	16.91 ± 2	4.41 ± 0.8	5.36 ± 0.5	n.q
Total DOC (%)	100%	11.2 ± 0.3%	88.8 ± 0.06%	2.2 ± 0.003%	55 ± 0.06%	14.3 ± 0.26%	17.4 ± 0.02%	-

n.q = non-quantifiable.

**Table 4:** Micro-pollutants in the ROC (ng/L) [26,36,37,38,39,40].

Micro-Pollutants	LOQ (ng/L)	Molecular Weight (MW) (g/mol)	Log K_ow_ (pH 7)	Charge	ROC (ng/L)
Atenolol	5	266	0.16	+	486 ± 20
Paracetamol	5	151	0.46		75 ± 30
Sulfamethoxazole	5	253	0.89	−	93 ± 45
Caffeine	10	194	−0.07	0	1095 ± 24
Trimethoprim	5	290	0.91	+/0	912 ± 42
TCEP ii	10	250	1.44		201 ± 28
Carbamazepine	5	236	2.45	0	2175 ± 60
Fluoxetine	5	309	4.10	+	44 ± 3
Clozapine	5	326	3.23	+	63.2 ± 5.2
Amtriptyline	5	277	4.92	+	40 ± 5
N,N-diethy1-3-methylbenzamide	5	191	1.96		72 ± 2.2
Primidone	5	218	0.91	−	26.5 ± 0.5
Verapamil	5	454	3.79	+	74.5 ± 8
Simazine	5	201	2.18	0	74.5 ± 5.5
Ketoprofen	5	254	3.12	−	260 ± 75
Naproxen	5	230	3.18	−	352 ± 69
Gemfibrozil	5	250	4.77	−	320 ± 42
Triclosan	5	290	5.34	0	181 ± 30
Diclofenac	5	296	4.51	−	324 ± 14
Triclocarban	10	316	4.90	0	147.5 ± 12
Diuron	5	233	2.68	0	318 ± 42

**Table 5:** Removal of Organic fractions by Purolite^®^ A502PS (Dosages 10 g/L and 20 g/L).

Purolite^®^ A502PS	DOC	Hydophobics	Hydrophilics	Biopolymers	Humics	Building Blocks	LMWs Neutrals
10 g/L	18,228 ± 112 (44.4 ± 0.27%)	3927 ± 50 (24.5 ± 0.3%)	14,301 ± 150 (48.1 ± 0.5%)	1256 ± 151 (−29.6 ± 3.5%)	7510 ± 410 (48.2 ± 2.6%)	1139 ± 65 (83.5 ± 4.77%)	4395 ± 50 (15 ± 0.17%)
20 g/L	15,363 ± 163 (53.1 ± 0.56%)	3305 ± 102 (36.5 ± 3.1%)	12,058 ± 212 (56.2 ± 0.9%)	1165 ± 71 (−20.2 ± 1.2%)	2665 ± 258 (81.2 ± 7.8%)	3785 ± 168 (45.2 ± 2%)	4441 (14.1 ± 0.04%)

**Table 6:** Removal of micro-pollutants using the MF–IEX hybrid system [60,61].

Micro-Pollutants	LOQ	Log Kow	Charge	MW (g/mol)	Raw ROC_1_	PUR 5 g/L	Removal (%)	Raw ROC_2_	PUR 20 g/L	Removal (%)
Atenolol	5	0.16	+	266	466 ± 12	114 ± 3	76 ± 1.1	506 ± 11	34 ± 4	93 ± 0.2
Paracetamol	5	0.49	−	151	114 ± 9	13 ± 1	88 ± 3	36 ± 2	<5	>86
Sulfamethoxazole	5	0.89	−	253	144 ± 18	11 ± 2	93 ± 2.5	42 ± 6	7 ± 2	84 ± 1
Caffeine	10	−0.07	0	194	1410 ± 116	97 ± 4	93 ± 0.5	717 ± 25	36 ± 4	95 ± 0.5
Trimethoprim	5	0.91	+/0	290	974 ± 50	149 ± 5	85 ± 1.5	852 ± 12	13 ± 2	98 ± 0.2
TCEP ii	10	1.44		250	229 ± 22	77 ± 2	66 ± 2	162 ± 9	29 ± 4	82 ± 1
Carbamazepine	5	2.45	0	236	2240 ± 145	386 ± 12	83 ± 1	2110 ± 115	40 ± 5	98 ± 0.5
Fluoxetine	5	4.10	+	309	47 ± 2	6 ± 1	87 ± 0.5	41 ± 5	5	88 ± 1
clozapine	5	3.53		326	68 ± 4	20 ± 4	71 ± 0.4	59 ± 6	<5	>92
amtriptyline	5	4.92	+	277	45 ± 8	5 ± 0.5	89 ± 0.2	35 ± 2	<5	>86
DEET	5	2.42		191	68 ± 12	13 ± 2	81 ± 2	74 ± 4	6 ± 1	92 ± 2
primidone	5	0.91	−	218	26 ± 5	5 ± 2	82 ± 1	27 ± 2	<5	>81
Verapamil	5	3.79	+	454	83 ± 4	6 ± 2	93 ± 1	66 ± 4	<5	>92
Simazine	5	2.18	0	201	80 ± 8	14 ± 4	83 ± 2	69 ± 4	<5	>93
Ketoprofen	5	3.12	−	254	377 ± 12	35 ± 2	91 ± 1.5	142 ± 6	<5	>96
Naproxen	5	3.18	−	230	443 ± 24	46 ± 6	90 ± 0.5	261 ± 8	5	98 ± 0.5
Gemfibrozil	5	4.77	−	250	344 ± 10	80 ± 4	77 ± 4	285 ± 12	9 ± 2	97 ± 1
Triclosan	5	5.34	0	290	211 ± 9	47 ± 5	78 ± 2	151 ± 11	19 ± 5	87 ± 2
Diclofenac	5	4.51	−	296	337 ± 14	117 ± 6	65 ± 2.5	310 ± 12	12 ± 5	96 ± 0.5
Triclocarban	10	4.90	0	316	162 ± 6	19 ± 4	88 ± 2	133 ± 12	15 ± 2	89 ± 2
Diuron	5	2.68	0	233	381 ± 7	29 ± 6	92 ± 0.2	256 ± 18	<5	>98

## Data Availability

Not applicable.

## Supplementary Materials

The following supporting information can be downloaded at: https://www.mdpi.com/article/10.3390/membranes13020136/s1, Figure S1: Five major EEM fluorescence regions (plotted according to Chen et al., 2003; Figure S2: Sample LC-OCD spectra showing different organic fractions; Table S1: Five major regions in EEM fluorescence spectra (28).
