# Supplementary material for: Removal of Organics with Ion-Exchange Resins (IEX) from Reverse Osmosis Concentrate
Source: Membranes (Basel). 2023 Jan 20;13(2):136. doi: 10.3390/membranes13020136 (PMC9967736; doi:10.3390/membranes13020136)
Supplement: Supplementary file 1 [file membranes-13-00136-s001.zip › membranes-2143270-supplementary.pdf]

Supplementary Material

Removal of organics by Ion-exchange resins (IEX) from Reverse Osmosis Concentrate

Sukanyah Devaisy[1,2], Jaya Kandasamy[1*], Rupak Aryal[1], Md Abu Hasan Johir[1], Harsha Ratnaweera[3], Saravanamuthu Vigneswaran[1,3*]

[1] Faculty of Engineering, University of Technology Sydney (UTS), Broadway, NSW 2007, Australia
[2] Department of Bio-Science, Faculty of Applied Science, University of Vavuniya, Vavuniya 43000, Sri Lanka
[3] Faculty of Sciences and Technology (RealTek), Norwegian University of Life Sciences, NO-1432 Ås, Norway
* Correspondence: jaya.kandasamy@uts.edu.au (J.K.); saravanamuth.vigneswaran@uts.edu.au (S.V.)

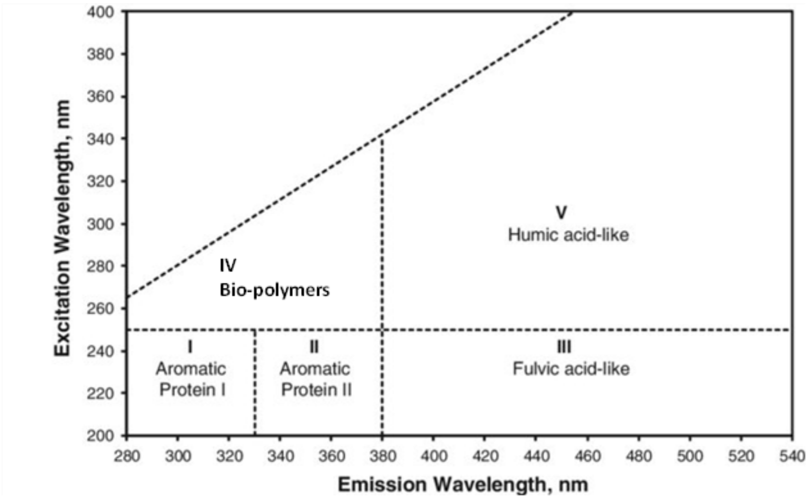

Figure S1. Five major EEM fluorescence regions (adapted from Chen et al. (28))

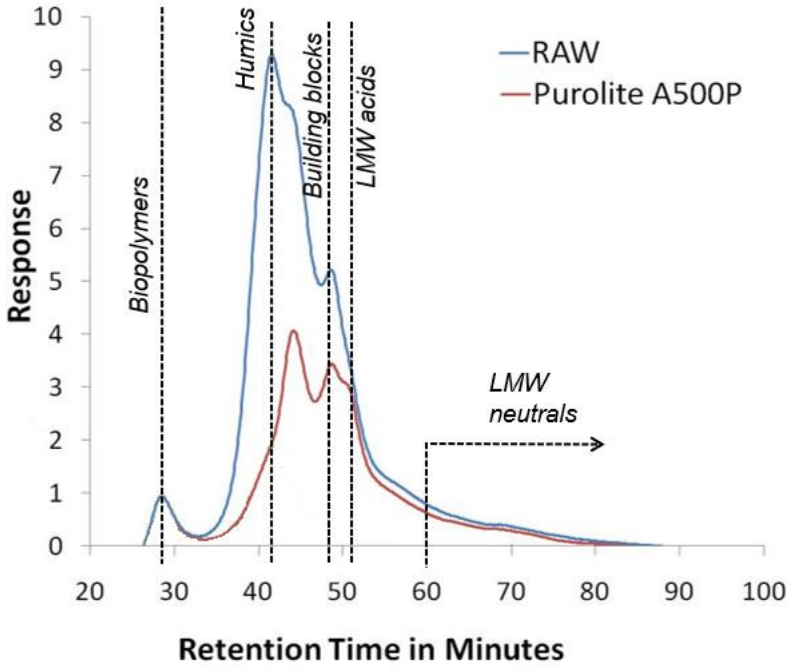

Figure 2. Sample LC OCD spectra showing different organic fractions

Table S1. Five major regions in EEM fluorescence spectra (28)

Region	Chemical composition of organic matter
I (P1): Ex:Em 200-250:280-330	lower molecular weight tyrosine-like aromatic amino acids
II (P2): Ex:Em 200-250:330-380	low molecular weight aromatic proteins and BOD-type substances
III (FA): Ex:Em 200-250:380-500	fulvic acid type substances)
IV(SMP): Ex:Em 250-340:280-380	large molecular weight peptides and proteins (microorganism related by-products
V(HA): Ex:Em 250-500:380-500	humic acid type substances
